# Annual Address, 1897

**Published:** 1897-09

**Authors:** James Truman

**Affiliations:** Philadelphia, Pa.


### Annual Address, 1897.

BY PRESIDENT JAMES TRUMAN, D.D.S., PHILADELPHIA, PA.

Abstract of address read before American Dental Association, August, 1897.

The annual gathering of a body such as this constitutes an era in dentistry and should mean an advance along all lines of work. If this fails to be realized the year has been valueless—a period lost in the progress of the race.

The present meeting is one of great importance ; if the feeling be judged aright, we have reached the "parting of the ways," and the rising sun of another day already appears above the horizon illuminating the century with a golden halo, indicating brilliant but, as yet, unfathomable possibilities.

The work of the past year will compare favorably with that of those preceding it, although the spirit of activity is not abroad in the land. Our calling tends to physical depression and disinclination to that higher work so necessary for advancement. The physical slavery which practice entails is not conducive to active mental effort. The man of investigating mind becomes eventually not a practitioner, but a laboratory devotee. Hence, there has arisen a small class who are the prophets of the age, and these are they to whom we have learned to look for the new ideas which mark progress.

The practical side of dentistry has not exhibited any features during the year worthy of special comment. The literature of this portion of our work has, however, been greatly enriched by several notable productions. While there has been little absolutely new, there has been marked improvement upon older methods of work.

Cataphoresis is gradually finding its true level ; its adoption has been phenomenal, and this constitutes its greatest danger, for the unwise rush in and attempt the impossible, forgetting that pain is the sentinel upon the advanced picket line and our ever-present warning of danger. The use of this method to the best good of the patient means a high order of anatomical knowledge, as far as the teeth are concerned, and this needs to be more and more impressed upon the dental mind.

The work on the scientific side of dentistry has received fresh impulse through the labors of Black, Andrews and Williams. The most brilliant exponent of this class of workers, Dr. W. D. Miller, is physically suffering from the great and exhaustive labors to which he has subjected himself. All here assembled will send back to him the expressed desire that he may know a full restoration to health and activity.

The near approach to the close of the nineteenth century naturally causes a retrospection. What have we stored up in the past one hundred years?

The first real step in advance dates back a little further than the century—to the introduction of porcelain teeth, by Chemaut, of France. Then came pulp devitalization by arsenic; pulp canal filling; the discovery of anæsthesia; the introduction of cohesive gold; the adoption of the mallet; the various forms of dental engines; the rubber dam, and finally the use of electricity as a motive power and as an obtundent. These are the most prominent and marked stages, each aiding to carry us forward to our present.

The crowning glory of the century, next to that of the discovery of anæsthesia, has been the solution of the problem of the ages—caries of the teeth.

The progress in dental education is unexampled in professional educational work; it has not only reached an equality with that of general medicine, but, in some respects, has led that venerable mother into paths she had not thought of treading.

To the National Association of Dental Faculties we owe all of the advances made in dental education in the United States, and we may look forward with truthful confidence that its work in the future will maintain the high record it has earned in the past.

The natural and final result of advanced thinking and acting is law—in a professional sense, the embodiment of orderly rules of conduct; hence the dental laws in every State, and the National Association of Dental Examiners.

Antagonism has been engendered by the unwise multiplication of statutes, and as the laws at present stand they are a dangerous obstacle in the path of professional progress and promise to be the one blot upon the otherwise fair fame of the century in dental work.

The aim should be for a unity of effort with the least friction of State with State, with a positive recognition that the decree of one State should, in this matter, be a law for every citizen of the United States. The Constitution of the United States expressly declares (Art. IV., Sec. 2.): "The citizens of each State shall be entitled to all privileges and immunities of citizens in the several States." It is, therefore clear, that a law passed, depriving a citizen who has been declared legally entitled to practice in any State from the privileges of registration in another State, is unconstitutional, and it is difficult to understand how the Supreme Court of the United States could decide otherwise if a case should be carried before it for adjudication.

In many of the States the members of the Board owe their places to political preference. This association should use its powerful influence to have all places on the boards filled upon the recommendation of State Dental Societies or by bodies having similar powers.

In regard to the contemplated reorganization of the dental associations, Dr. Truman said:

Reorganization to be effective must be based upon an entire change of methods. Without attempting a solution of this matter it may serve a purpose to point out what may be regarded as some things worthy of change in the old associations.

Dr. Truman then considered the relation of local societies to the national body, a national body being a necessary part of the organization of a profession. Local societies must be established upon a positive standard of entrance, delegates from these societies becoming, if they so desire, permanent members of the national organization and part of the controlling body. It will be difficult to change present methods in local bodies, but proper legislation in the national organization would, in time, effect much. The methods of work by sections has been an improvement over older systems. The system is intrinsically weak and can never effect any permanent good results. The establishment of permanent membership has been the deadly poison that has eaten out the life of the American Dental Association. It is an aristocracy band—not a prize for myth's work, but upon *ability to pay* the yearly fee and attendance at the annual meetings. Is it not time to originate a body distinct from all others, and which should embrace elements of strength rather than weakness? A national body should be the culmination of all below it. It should embrace the concentrated wisdom of all subordinate societies. The affiliating organizations should send to the national only such members as have given the profession satisfactory work. With this care in the selection of members, the national body would partake of the character of the higher scientific bodies of older civilization.

The regular practitioner of dentistry cannot work for the advance of his profession if it interferes with his daily work. The true investigator of problems finds practice irksome and is ready to burst the bonds which environ him for the more enjoyable fields of the mysterious unknown.

The time seems to have arrived when means should be found to set free these imprisoned students and make it possible for them to work independently of the cares of life.

When the new organization is perfected it is hoped the possibilities in this direction may be considered and a fund established looking to financial aid to those who are continually sacrificing themselves that the profession may make progress worthy of its high aspirations.

